# Integrated proteomics and metabolomics analysis of rat testis: Mechanism of arsenic-induced male reproductive toxicity

**DOI:** 10.1038/srep32518

**Published:** 2016-09-02

**Authors:** Qingyu Huang, Lianzhong Luo, Ambreen Alamdar, Jie Zhang, Liangpo Liu, Meiping Tian, Syed Ali Musstjab Akber Shah Eqani, Heqing Shen

**Affiliations:** 1 Key Laboratory of Urban Environment and Health, Institute of Urban Environment, Chinese Academy of Sciences, Xiamen 361021, PR China; 2 Ningbo Urban Environment Observation and Research Station-NUEORS, Chinese Academy of Sciences, Ningbo 315800, PR China; 3 Department of Pharmacy, Xiamen Medical College, Xiamen 361008, PR China

## Abstract

Arsenic is a widespread metalloid in environment, whose exposure has been associated with a broad spectrum of toxic effects. However, a global view of arsenic-induced male reproductive toxicity is still lack, and the underlying mechanisms remain largely unclear. Our results revealed that arsenic exposure decreased testosterone level and reduced sperm quality in rats. By conducting an integrated proteomics and metabolomics analysis, the present study aims to investigate the global influence of arsenic exposure on the proteome and metabolome in rat testis. The abundance of 70 proteins (36 up-regulated and 34 down-regulated) and 13 metabolites (8 increased and 5 decreased) were found to be significantly altered by arsenic treatment. Among these, 19 proteins and 2 metabolites were specifically related to male reproductive system development and function, including spermatogenesis, sperm function and fertilization, fertility, internal genitalia development, and mating behavior. It is further proposed that arsenic mainly impaired spermatogenesis and fertilization via aberrant modulation of these male reproduction-related proteins and metabolites, which may be mediated by the ERK/AKT/NF-κB-dependent signaling pathway. Overall, these findings will aid our understanding of the mechanisms responsible for arsenic-induced male reproductive toxicity, and from such studies useful biomarkers indicative of arsenic exposure could be discovered.

Arsenic (As) is a toxic metalloid widespread in natural environment. Humans can be exposed to arsenic through environmental, agricultural, and occupational routes, among which contaminated drinking water poses one of the major threats to human health. It has been estimated that approximately 19.6 million people are exposed to arsenic via consumption of groundwater in China^1^. There is increasing epidemiological evidence indicating that exposure to arsenic was associated with skin pathologies, chronic obstructive pulmonary disease, cardiovascular diseases, diabetes, hepatitis, developmental neurotoxicity, adverse birth outcomes, reduced sperm quality and genotoxicity^2^,^3^,^4^,^5^,^6^,^7^. In addition, arsenic was classified as a carcinogen, which has been linked with the onset and progression of tumors in lung, liver, kidney, skin and bladder^2^,^8^.

At present, the toxic effects of arsenic exposure on the male reproductive system have been documented. Arsenic was demonstrated to impair the development of reproductive organs, inhibit steroidogenesis and reduce sperm quality, which may result in male infertility. These toxic effects are frequently attributed to the oxidative stress induced by excess reactive oxygen species (ROS), which causes lipids, proteins, and DNA damage^9^,^10^,^11^. Jana *et al*. pointed out that arsenic may cause male reproductive toxicity through an estrogenic mode of action^12^. Furthermore, inhibition of androgen receptor transcriptional activity was considered to play an important role in arsenic-induced male infertility^13^. Epidemiological survey also showed that arsenic exposure reduces human semen quality and increases the risk of prostate carcinogenesis^14^,^15^. By analyzing the differential urinary metabolome of infertile men in comparison to the control, Shen *et al*. found that arsenic may exert toxicity via oxidative stress and sexual hormone disruption, as indicated by related metabolic biomarkers^16^. However, these studies payed attention to single or several effects of arsenic, and it is imperative that a global view of arsenic-induced male reproductive toxicity be further clarified. Furthermore, the involved toxic mechanisms are still far from being completely understood.

Proteins are the primary effector molecules of all living systems, and any adaptive responses to exotic stresses will be reflected by alterations in protein activity or content^17^. Metabolic patterns, the endpoints of enzyme (protein) actions, are the final consequence of biological function, and they can directly indicate aberrant physiological status. ‘Omics’ technologies, including proteomics and metabolomics, which provide information on global profile, are therefore regarded as more powerful tools to investigate toxic responses to environmental pollutant exposure than conventional endpoint bioassays^18^,^19^,^20^. In addition, integration of proteomic and metabolomic profiles may provide greater reliability in interpreting metabolic alterations caused by certain proteins and allow for further elucidation of the toxicological effects and mechanisms^21^. There have been extensive reports addressing the global effects of arsenic exposure on liver, bladder, kidney, skin, serum and urine using single proteomics or metabolomics approach^22^,^23^,^24^,^25^,^26^,^27^. To date, however, integrated proteomic and metabolic signatures are still rarely characterized for male reproductive toxicity induced by arsenic.

In the present study, high-throughput label-free quantitative proteomics and UPLC/MS-based metabolomics approaches were employed to investigate the alterations of proteomic and metabolomic profiles in rat testis following arsenic treatment. Moreover, the signaling pathways involved in arsenic action were further analyzed. The results will be helpful to gain comprehensive insights into the mechanisms regarding arsenic-induced male reproductive toxicity, and to develop potential biomarkers for the health risk assessment of environmental arsenic exposure.

## Materials and Methods

### Animals and arsenic treatment

Animal experiments were approved by the Institutional Animal Ethics Committee of Institute of Urban Environment, Chinese Academy of Sciences, and all experiments were carried out in accordance with the approved guidelines. Sprague-Dawley (SD) male rats (4 weeks of age, 80 g) were sourced from SLAC Laboratory Animal (Shanghai, China). The rats were housed in plastic cages for natural diet at controlled conditions: room temperature of 23 ± 2 °C, relative humidity of 45 ± 10%, and a 12 h light-dark cycle. After acclamation for one week, 40 rats were randomly distributed into four groups, each group containing 10 rats. Three groups of animals were administered with 1 mg/L, 5 mg/L and 25 mg/L of sodium arsenite (SA) via drinking water *ad libitum*, respectively; the control group received deionized water by the same way. After the exposure of 6 months, rats were sacrificed following anesthesia. Blood was quickly collected, and testes were carefully dissected and stored in liquid nitrogen until further processing.

The range of arsenic concentration found in natural waters around the world varied from <0.0005 to >5 mg/L^28^, and there is a report that in West Bengal, India, people were exposed to arsenic at a maximum concentration of 3.7 mg/L in groundwater^29^. In this study, 25 mg/L of SA was used as high dose for rat exposure, which was approximately equivalent to 14.4 mg/L of arsenic, and was 2.3 mg/L for human^30^. In addition, 25 mg/L of SA in drinking water (equivalent to 4 mg/kg bw) is about 1/10 of the oral median lethal dose (LD_50_) of SA in rats (41 mg/kg bw)^31^. Taken together, the doses used in current study relate to arsenic exposure level for human as well as to LD_50_ of SA in rats, and therefore, are considered to be environment relevant and physiologically significant.

### Quantification of total arsenic in rat serum and testis

Rat blood was centrifuged at 3500 × *g* for 10 min, and the supernatant was collected as serum. For total arsenic analysis, 500 μL of serum and 50 mg of testis tissue were digested with 1 mL HNO_3_ (65%) overnight, respectively. A further 1 mL H_2_O_2_ (30%) was added and the mixture was transferred into a microwave digest tube. In the microwave oven, the digested mixture was exposed to 800 W for 10 min under 120 °C and 30 min under 170 °C. Tubes were then cooled down to room temperature, and the volumes were raised up to 5 mL with deionized water. The total arsenic levels of the samples were measured using an Agilent 7500cx inductively-coupled plasma mass spectrometry (ICP-MS, Santa Clara, CA, USA). All samples were analyzed in batches along with blanks and standards towards a standard calibration curve.

### Sperm counts, sperm motility and hormones analysis

For sperm sampling, the rat cauda epididymis was placed in PBS, cut into small pieces, and cultured in DMEM medium supplemented with 10% fetal bovine serum at 37 °C for 30 min to allow the sperm to swim up. The sperm suspension was then collected and sperm counts were calculated by a haemocytometer under light microscope. The sperm suspension was diluted with 5 volume of the same medium and incubated for at 37 °C 15 min. The upper and the lower suspension were then separately collected, and the sperm count was assessed. The percentage of sperm motility was calculated using the number of motile sperm over the total number. The contents of testosterone and estradiol in rat serum were detected by radioimmunoassay (RIA) using commercial kits as reported previously^32^.

### Label-free quantitative proteomics analysis

Details of protein sample preparation, tryptic digestion and proteomic profiling acquisition were described in Supporting Information. The acquired LC-MS/MS data were analyzed using the MaxQuant software (http://maxquant.org/, version 1.5.2.8) for protein identification and quantification^33^. The Andromeda search engine was used for matching the peak lists against a concatenated forward and reverse database including the complete UniProt-SwissProt rat database (reviewed, with a total of 9638 entries) and the standard MaxQuant contaminant database. The following settings were chosen for the MaxQuant software analysis: Carbamidomethyl (C) was set as a fixed modification; Oxidation of methionine and N-terminal acetylation were set as variable modifications. Peptide tolerance was set to 20 ppm for the first and 4.5 ppm for the main search. The maximum number of peptide modifications was set to 5. Trypsin/P was selected as the digestive enzyme and the maximum number of missed cleavages was 2. Peptide and protein false discovery rate (FDR) were set to 0.01. The minimum number of peptide counts was 1 and peptide intensity threshold was set to 500. Quantification was achieved using the LFQ (Label-Free Quantification) algorithms. Protein identification and quantification data were obtained from an output file named proteinGroups (txt format). Protein fold changes were calculated by normalizing the LFQ intensity of treatment groups to that of control (treatment/control ratio, control = 1). The mass spectrometry proteomics data have been deposited to the ProteomeXchange Consortium (http://proteomecentral.proteomexchange.org) via the PRIDE partner repository with the dataset identifier PXD004364.

### Metabolomics analysis

Details of sample preparation, metabolic profiling acquisition, data processing and quality control procedure were described in Supporting Information. The processed feature tables were then Pareto-scaled and submitted to SIMCA-P V11.5 software (Umetrics, Uppsala, Sweden) for multivariate statistical analysis. Principal component analysis (PCA) was first performed to discover intrinsic treatment-related clusters within the datasets. Following this, partial least-squares discriminant analysis (PLS-DA) was used to improve separation among the groups and screen biomarkers. A cross-validation procedure and testing with 999 permutations were performed to avoid overfitting of the supervised PLS-DA model. Variable importance in projection (VIP) represents the extracted variables’ ability to discriminate between different doses, and the variables with VIP values greater than 1.0 were included in the preset of biomarkers. Metabolite identification based on UPLC-MS data was carried out by searching Human Metabolome Database (HMDB, http://www.hmdb.ca) based on accurate mass measurement. An accepted mass difference of 50 mDa was set during the search. Furthermore, the UPLC/MS/MS product ion spectrum of metabolites was matched with the MS spectra available in HMDB to confirm a positive identification (Table S1).

### Molecular network analysis

To model the signaling networks being affected by arsenic in rat testis, the ID numbers and fold changes of arsenic-regulated proteins and metabolites were uploaded and subjected to network analysis using the Ingenuity Pathways Analysis (IPA) software (http://www.ingenuity.com). IPA computed a score for each of the possible networks in accordance to the fit homology to the input molecules. This score is derived from a *p*-value and indicates the probability of the input molecules in a given network to coexist as a result of random chance (*p*-score = −log_10_ *p*-value). Network scores of ≥2 has a >95% confidence of not being randomly generated.

### Quantitative real-time PCR

Total RNAs were extracted using the RNeasy^®^ Mini Kit (Qiagen) from rat testes. Reverse transcription of cDNA synthesis was performed with 1 μg of RNA using PrimeScript^®^ RT reagent Kit (Takara, Japan). Real-time PCR was carried out in a 20 μL reaction mixture using SYBR Green Master Mix reagents (Roche, USA) on a LightCycler^®^ 480II real-time PCR system (Roche, USA) following the manufacturer’s protocol (95 °C for 10 min followed by 40 cycles at 95 °C for 15 s, and 60 °C for 1 min). Gene expression levels were normalized to β-Actin gene expression. All primer sets are described in Table S2. Three replicates for each sample were performed. The fold changes (treated/control) of tested genes were analyzed by the 2^−ΔΔCT^ method.

### Western blotting

Testis protein samples (100 μg) were separated by 12% SDS-PAGE. After electrophoresis, the resolved proteins were transferred to PVDF membrane (Roche). The membrane was then blocked for 1 h in TBST (50 mM Tris-HCl, pH 7.6, 150 mM NaCl and 0.1% Tween 20) containing 5% (w/v) nonfat milk, and incubated with primary antibodies of anti-ERK1/2, anti-AKT, anti-Phospho-ERK1/2 (Thr202/Tyr204), anti-Phospho-AKT (Ser473) and anti-β-Actin (Cell Signaling Technology, 1:1000 dilution) overnight. The membrane was washed three times with TBST and incubated with secondary antibody HRP-conjugated goat anti-rabbit IgG (Pierce, 1:10000 dilution) for 1 h. The membrane was again washed three times with TBST, and the blots were developed using ECL. The signals from target protein bands were normalized against the β-Actin contents.

### Statistical analysis

The data are all expressed as mean ± standard deviation (SD), and the statistical analysis was performed with SPSS software (Version 18.0). Significant differences among multiple groups were determined using a one-way analysis of variance (ANOVA) followed by LSD *post-hoc* test. Probabilities of *p* < 0.05 were considered as statistically significant.

## Results

### Concentration of arsenic in rat serum and testis

**Figure 1:**
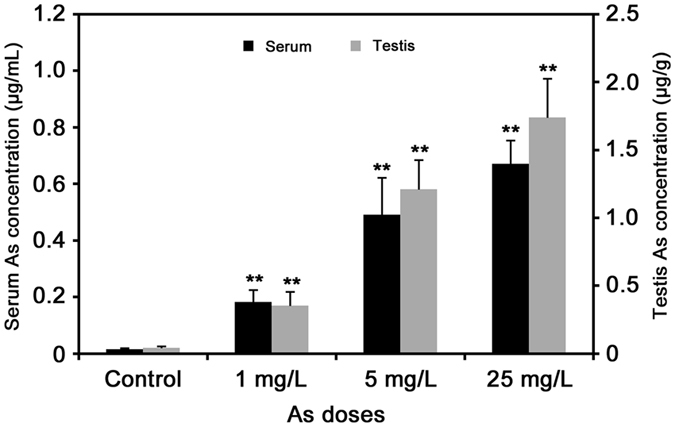
Concentrations of total arsenic in rat serum and testis measured by ICP-MS. The values were expressed as mean ± SD, ^*^p < 0.05, ^**^p < 0.01.

During the whole exposure period, arsenic did not cause any rat mortality. In addition, the body weight (BW), testis weight (TW) and testicular coefficient (TW/BW) of rats were not significantly altered by arsenic exposure (*p* > 0.05, Table S3). The concentrations of arsenic in rat serum and testis were further determined by ICP-MS. As shown in Fig. 1, the arsenic level in serum and testis of rat in the three treated groups were significantly higher than those in the control (*p* < 0.01), which increase in a typical dose-dependent manner. The arsenic concentration in serum ranged from 0.18 to 0.67 μg/mL, while it ranged from 0.35 to 1.74 μg/g in testis tissue. These results suggest that arsenic can pass through the blood-testis barrier and accumulate in rat testis, which may subsequently induce a variety of adverse effects on male reproduction.

### Effects of arsenic exposure on sperm quality and hormone production

**Figure 2:**
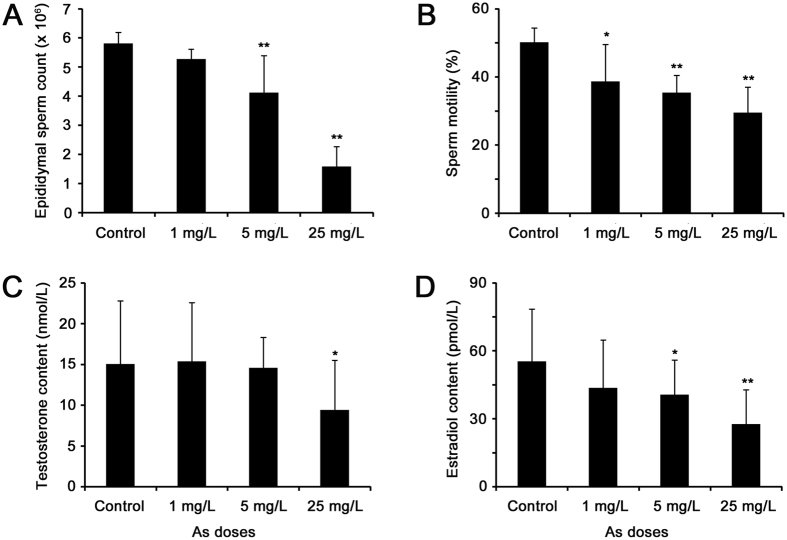
Effects of arsenic exposure on sperm counts (**A**), sperm motility (**B**), testosterone (**C**) and estradiol (**D**) contents in rats. The values were expressed as mean ± SD, ^*^p < 0.05, ^**^p < 0.01.

To investigate the toxic effects of arsenic on male reproduction, we analyzed the sperm quality and hormone levels in rat. Sperm counts and sperm motility were both significantly reduced in arsenic-treated rats compared with the control (Fig. 2A,B). Testicular Leydig cells play a vital role in maintaining proper levels of male hormones, which are essential for male reproductive function, including spermatogenesis. The contents of testosterone and its product estradiol were also observed to decrease in rat serum after arsenic treatment (Fig. 2C,D). Thus, it is indicated that arsenic inhibited testosterone synthesis, which then impaired spermatogenesis and produced low-quality sperm in rats.

### Identification of arsenic-regulated proteins in rat testis

To characterize the expression changes of proteins response to arsenic exposure, the label-free quantitative proteomics technology was used to analyze the rat testis proteome of control and three treated groups (1, 5 and 25 mg/L). Overall, a total of 2382 proteins were identified with a FDR less than 1% at the peptide and protein level, and 703 proteins were quantifiable in all the 12 samples (four groups with three technical replicates each, Table S4). Of these proteins, only 70 proteins showed significant expression differences (*p* < 0.05, fold change ≥1.2 at least in 25 mg/L treatment group)^32^, indicating no obvious expression changes for most of the identified proteins in arsenic-treated rat testis, and 36 proteins were up-regulated whereas 34 were down-regulated (Table S5).

In order to obtain an overview of the effects of arsenic exposure on rat testis, the differential proteins were categorized according to their biological processes by searching the Gene Ontology database (http://geneontology.org/). Especially, 19 proteins (27%) were associated with reproductive process (Table 1), and others were mainly involved in cellular process, stimulus response, cellular component assembly, protein organization and energy metabolism (Fig. S1).

### Differential metabolic profiles in arsenic-treated rat testis

PCA was initially performed on the LC-MS datasets to visualize general clustering trends among the observations. However, there did not appear to be clear segregation of the metabolomic profiles of the arsenic-treated groups from the control for both positive and negative ion mode (data not shown). A supervised PLS-DA model was further used to identify the differences among different groups, and the models were successfully established for positive mode but not for negative one, indicating little differences among the metabolites obtained under negative mode. Therefore, the subsequent results are only described for positive mode.

**Figure 3:**
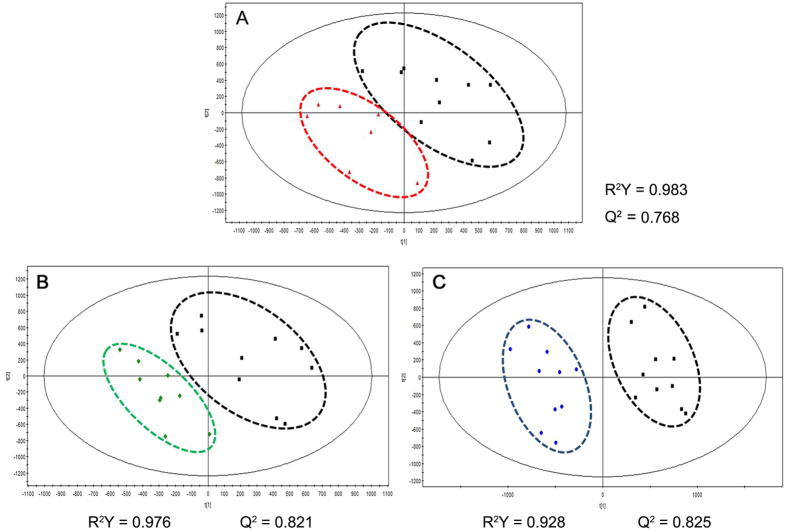
Scoring plots of metabolites from rat testis with PLS-DA model under positive ion mode. 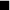
 Control; 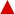 1 mg/L; 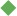 5 mg/L; 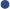 25 mg/L

As can be seen in Fig. 3, the three arsenic-treated groups were clearly discriminated from the control group by the first two components based on PLS-DA models, indicating a faithful representation of the data and a good predictive ability of the model. In addition, the PLS-DA model was validated by a permutation test (999 random permutations), and no overfitting of the data was observed (Fig. S2). The results suggested that arsenic exposure led to significant metabolic alterations in rat testis.

### Metabolic biomarker screening and identification

Extracted variables that contributed the most in group distinction were chosen as the biomarkers for arsenic exposure. Strict criteria were adopted in the screening: (1) the variables with a VIP value > 1 were brought into the superset of biomarkers; 2) the candidates with negative jack-knifed confidence intervals were removed (Fig. S3); (3) the candidates were retained in all groups; and (4) the difference of candidate level (relative peak area) was statistically significant (*p* < 0.05, Mann−Whitney U test) between the control and treatment groups. Following these criteria, 13 altered metabolites were identified and considered as potential biomarkers (Table 2), among which 8 metabolites were increased while 5 were decreased by arsenic treatment.

The metabolic pathways involved in the differential metabolites were analyzed using the MetaboAnalyst 2.0 software (http://www.metaboanalyst.ca). As a result, the software generated 4 metabolic pathways with a *p*-value < 0.05, which were considered to be significantly associated with arsenic-induced metabolic changes. These 4 pathways were characterized as aminoacyl-tRNA biosynthesis, phenylalanine, tyrosine and tryptophan biosynthesis, phenylalanine metabolism, as well as ubiquinone and other terpenoid-quinone biosynthesis (Table S6). In brief, amino acid biosynthesis and metabolism seems to be the major metabolic pathways disturbed by arsenic in rat testis.

### Molecular networks involved in arsenic treatment

**Figure 4:**
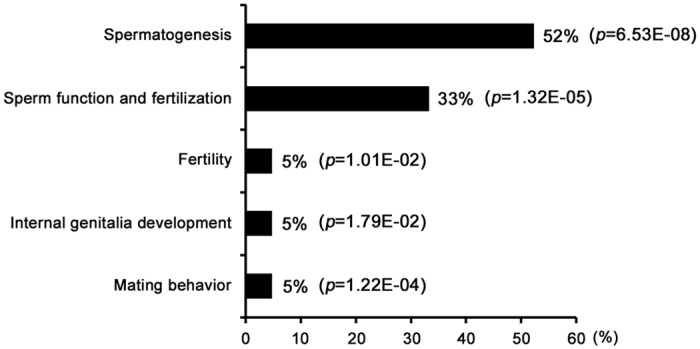
Functional category of the differential proteins and metabolites related to male reproductive system development and function in rat testis exposed to arsenic.

**Figure 5:**
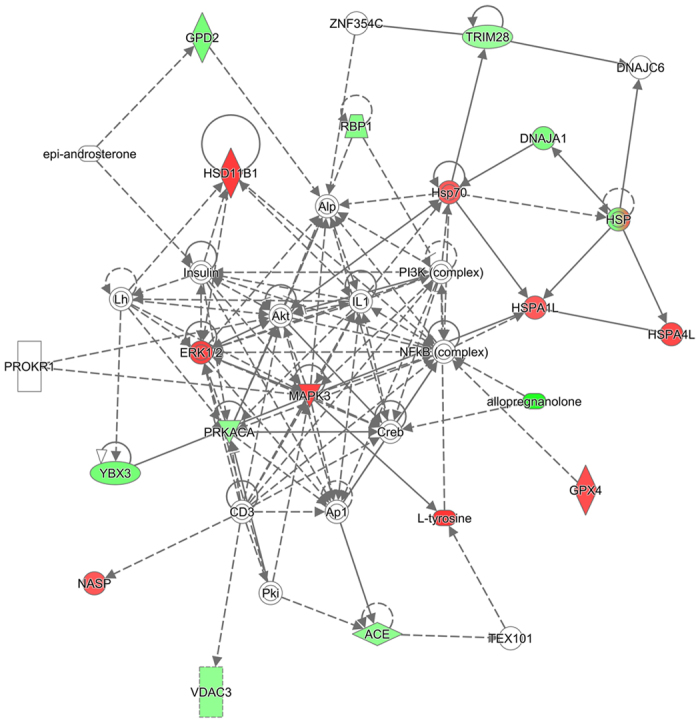
Network analysis of the differential proteins and metabolites performed using the IPA software. The network with the highest score that describes molecules involved in reproductive system development and function, organ morphology, as well as organismal injury and abnormalities is presented. Molecules are represented as nodes. Nodes in red represent up-regulated molecules, while nodes in green represent down-regulated ones. Molecules represented by white nodes were not observed. Solid lines indicate direct interactions or regulation, while dashed lines indicate indirect effects mediated by additional molecules.

To investigate whether the differential proteins and metabolites interacted biologically, IPA software was used to generate network diagrams to elucidate signaling pathways impacted by arsenic in rat testis. Nineteen proteins involved in reproductive process (Table 1) and 13 modulated metabolites (Table 2) were imported to the IPA module, and IPA mapped 19 proteins and 10 metabolites, which were eligible for network generation. As shown in Fig. 4, 19 proteins and 2 metabolites (tyrosine and allopregnanolone) were associated with male reproductive system development and function (*p* < 0.05), including spermatogenesis (52%), sperm function and fertilization (33%), fertility (5%), internal genitalia development (5%) and mating behavior (5%). Four networks gaining a score of ≥2, which correspond to identification at 95% confidence were built by IPA (Table S7). The network that received the highest score (a score of 45) was a network (Network 1) with 16 “focus molecules”, and the top three of functions associated with the network were classified as reproductive system development and function, organ morphology, and organismal injury and abnormalities (Fig. 5). Moreover, it seems that ERK1/2, AKT and NF-κB are the key regulators of this network, which may suggest their critical roles in arsenic-induced male reproductive toxicity.

### Arsenic-induced male reproductive toxicity is involved in ERK/AKT/NF-κB signaling pathway

**Figure 6:**
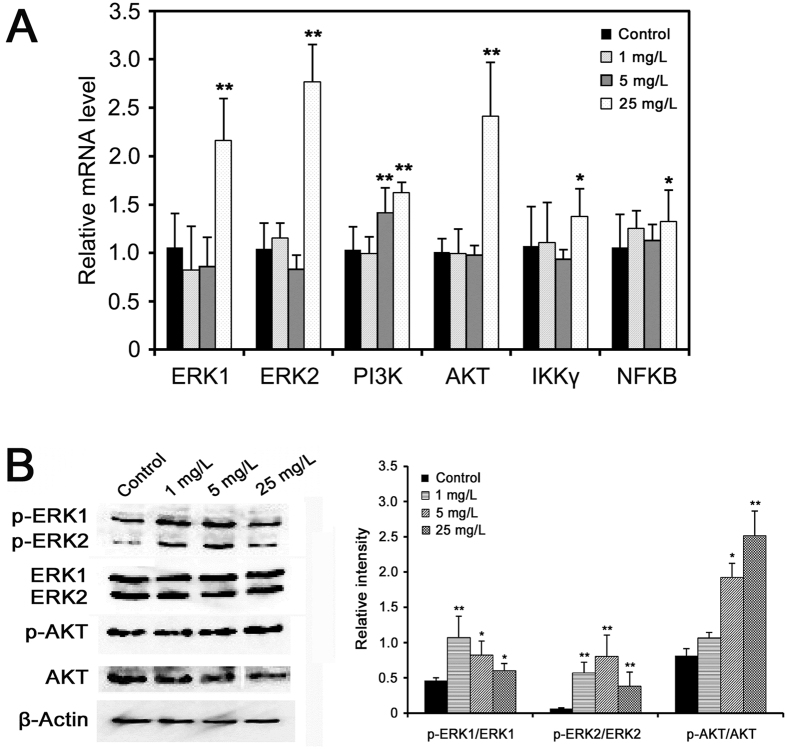
(**A**) Quantitative real-time PCR analysis of the expression levels of key genes involved in ERK/AKT/NF-κB pathway in rat testis exposed to arsenic. The data of treatments were calibrated to the control values (control = 1). (**B**) Testis protein was separated by SDS-PAGE and probed with the indicated antibodies. β-Actin was measured as the loading control and used for data normalization. The relative band intensity of the target protein was normalized to β-Actin level, and the content of phosphorylated-ERK1/ERK2/AKT was relative to the level of ERK1/ERK2/AKT, respectively. Values were all expressed as mean ± SD, ^*^p < 0.05, ^**^p < 0.01.

To establish a link between arsenic-induced male reproductive toxicity and ERK, AKT and NF-κB signaling pathways, we further determine the expression levels of several key genes involved in ERK/AKT/NF-κB pathway in rat testis following arsenic exposure. As a result, the mRNA levels of ERK1, ERK2, PI3K, AKT, IKKγ, and NFKB were all significantly up-regulated (Fig. 6A). In addition, the protein levels of phosphorylated ERK1/2 and AKT were examined using Western blotting. As can be seen in Fig. 6B, the levels of phosphorylated ERK1 (p-ERK1/ERK1), ERK2 (p-ERK2/ERK2) and AKT (p-AKT/AKT) were all significantly elevated in rat testis exposed to arsenic, indicating that ERK/AKT/NF-κB signaling was activated by arsenic treatment. These results suggest that arsenic may induce male reproductive toxicity through ERK/AKT/NF-κB-dependent pathway.

## Discussion

Through identifying critical proteins, metabolites and the involved pathways in biological systems that are affected by and respond to environmental stresses using global profiling technologies, toxicoproteomics and toxicometabolomics are helpful to augment our understanding of the toxic mechanisms associated with arsenic exposure. Therefore, this study investigated the alterations of protein and metabolic profiles in rat testis exposed to arsenic by using an integrated proteomics and metabolomics approach. Nineteen differential proteins and two metabolites closely related to male reproduction were identified, and the disturbance of spermatogenesis and fertilization are suggested as major factors contributing to arsenic-mediated male reproductive toxicity.

### Arsenic impairs spermatogenesis in rat testis

Spermatogenesis is a complex process to produce mature spermatozoa, which is essential for male reproduction. Here, 5 up-regulated and 5 down-regulated proteins, as well as 1 decreased metabolite involved in spermatogenesis were responsive to arsenic treatment in rat testis. Glutathione peroxidase 4 (GPx4), an antioxidant enzyme, is the predominant selenoenzyme in testis and has been shown to be crucial for spermatogenesis^34^. Overexpression of GPx4 in mouse testis caused spermatogenetic defects, including primary spermatocyte apoptosis, haploid cell loss, and seminiferous epithelium disorganization^35^. Also, endocrine disrupting chemicals pose a detrimental effect on spermatogenesis via aberrant enhancement of GPX4 expression in rat testis^36^. Corticosteroid 11β-dehydrogenase isozyme 1 (11β-hydroxysteroid dehydrogenase, HSD11B1), the enzyme that catalyzes the conversion of inactive cortisone to biologically active cortisol, is located exclusively in Leydig cells of rat testis. HSD11B1 was suggested to play an important role in maintaining steroidogenesis by generating cortisol that involved in testosterone production^37^. Higher HSD11B1 activity has been associated with lower sperm count and higher level of morphologically abnormal spermatozoa^38^. Nuclear autoantigenic sperm protein (NASP) is a histone chaperone that binds H1 linker histones and has been proposed to transport them into the nucleus of dividing cells. Testicular NASP (tNASP) was demonstrated to be involved in cell cycle progression in spermatogenic cells, probably through an interaction with the Cdc2/cyclin B and Hsp70-2 complex^39^. And overexpression of tNASP was observed during androgen receptor blockade, when spermatocyte meiosis would likely be inhibited^40^. Calcium-binding and spermatid-specific protein 1 (CABS1) is a calcium-binding protein that specifically expressed in the elongate spermatids. It is involved in the extremely complex structural rearrangements occurring in haploid germ cells during spermatogenesis^41^. Heat shock 70 kDa protein 4-like (HSPA4L) belongs to the HSP110 heat shock protein family, and is expressed ubiquitously and predominantly in the testis. It was found that the number of mature sperm and sperm motility were drastically reduced in HSPA4L-dificient male mice due to higher levels of apoptotic germ cells^42^. In this study, the expression level of GPX4, HSD11B1, NASP and CABS1 were elevated, implying that spermatogenesis was impaired and low-quality sperm would be produced, which is supported by our findings that sperm number and motility were reduced in arsenic-treated rats (Fig. 2A,B). However, the up-regulation of HSPA4L may indicate a resistance to arsenic-induced germ cell apoptosis.

Scaffold attachment factor B1 (SAFB1) contains a transcriptional repression domain and can bind certain hormone receptors and repress their activity. Male SAFB1 null mice were infertile because of increased apoptosis of germ cells, Leydig cell hyperplasia and low testosterone level, which may be attributed to the reduction of circulating insulin-like growth factor 1 (IGF1) and loss of SAFB1-mediated repression of hormone receptors^43^. Transcriptional intermediary factor 1 (TIF1) β (also named KAP-1 or TRIM28) is a transcriptional co-repressor known to play key roles in spermatogenesis and early embryonic development. During spermatogenesis, TIF1β is preferentially associated with heterochromatin structures of Sertoli cells and round spermatids, as well as with meiotic chromosomes^44^. Herzog *et al*. observed that the absence of TIF1β leads to a clear defect of spermatogenesis characterized by the failure of spermatid release and testicular degeneration^45^. Retinols are required for the maintenance of normal spermatogenesis in mammalian testis, and prolonged deficiency of retinols results in spermatogenic arrest at preleptotene spermatocytes followed by extensive loss of germinal epithelium in rats. Retinol is delivered to the seminiferous tubules via a specific plasma transport protein, retinol-binding protein 1 (RBP1), which is mainly localized in Sertoli cells. As documented previously, RBP1 may be involved in the uptake of retinol into Sertoli cell and its esterification, as well as the transfer of retinol to developing germ cells^46^. DnaJ homolog subfamily A member 1 (DNAJA1) works similarly as a co-chaperone of Hsp70 s in protein folding and mitochondrial protein import. It was reported that loss of DNAJA1 in mice led to a severe defect of Sertoli cells in maintaining spermatogenesis, increase of androgen receptor (AR), and disruption of Sertoli-germ cell contact, indicating a critical role of DNAJA1 in spermatogenesis through AR-mediated signaling in Sertoli cells^47^. The Y-box protein family is recognized as one of the most evolutionarily conserved nucleic acid binding protein families. A remarkable decrease in protamine 2 transcription was seen when both the PAF-RE and Y-box binding protein 3 (YBX3, YB2) were deleted, which suggested that YBX3 is needed to activate protamine 2 transcription in postmeiotic male germ cells^48^. Allopregnanolone is the metabolite of progesterone by actions of 5α-reductase and 3α-HSD^49^. Since progesterone is a key intermediate of testosterone biosynthesis pathway, which is essential for maintaining spermatogenesis, it is tempting to conceive that arsenic-induced decrease of allopregnanolone level was ascribed to the reduction of progesterone, which would impair testosterone synthesis and followed spermatogenesis. Linking to the reduced testosterone and sperm quality (Fig. 2), we proposed that depletion of SAFB1, TRIM28, RBP1, DNAJA1, YBX3 and allopregnanolone would lead to abnormal testicular spermatogenesis because of germ cell deficiency and lower testosterone level in arsenic-exposed rats.

### Arsenic hinders fertilization of rat sperm

In animals, fertilization involves the fusion of a sperm with an ovum, which first creates a zygote and then leads to the development of an embryo. Regarding to this work, 6 down-regulated proteins and 1 increased metabolite were found to be associated with fertilization in arsenic-exposed rat. Voltage-dependent anion channel protein 3 (VDAC3) is an isoform of VDACs, which are present in the mitochondrial proteins of all eukaryotes^50^. It was shown that VDAC3 is localized on the acrosomal region and midpiece. Blocking VDAC3 significantly decreased acrosome reaction, tyrosine phosphorylation, capacitation and subsequent fertilization, suggesting the pivotal role of VDAC3 in male fertility^51^. cAMP-dependent protein kinase catalytic subunit alpha (PRKACA) is a serine/threonine kinase activated by cAMP, which then leads to downstream events including protein tyrosine phosphorylation. The inhibition of PRKACA was reported to abolish protein tyrosine phosphorylation signaling and ultimately inhibit sperm capacitation^52^. Glycerol-3-phosphate dehydrogenase 2 (GPD2) is one of the proteins that enables tyrosine phosphorylation during sperm capacitation. The enzymatic activity of GPD2 correlates positively with hyperactivation and acrosome reaction, which suggests the roles of GPD2 in sperm capacitation. GPD2 activity is required for ROS generation in mouse spermatozoa during capacitation, failing which, capacitation is impaired^53^. Tyrosine phosphorylation of proteins is one of the most common mechanisms through which several signal transduction pathways are adjusted in spermatozoa. It regulates various aspects of sperm function, such as motility, hyperactivation, capacitation, acrosome reaction and fertilization^54^,^55^. Thus, we suggest that arsenic-induced repression of VDAC3, PRKACA and GPD2 as well as the aberrant increase of L-tyrosine may disrupt the extent of protein tyrosine phosphorylation required for sperm capacitation, which then result in fertilization failure and male infertility.

Sperm acrosome membrane-associated protein 1 (SPACA1) is a membrane protein that localizes in the equatorial segment of spermatozoa in mammals and functions in sperm-egg fusion. Disruption of SPACA1 was found to lead to abnormal shaping of the sperm head (globozoospermia), which resulted in male mice infertile^56^. In addition, antibodies against recombinant SPACA1 also inhibited both the binding and the fusion of sperm to zona-free eggs^57^. Angiotensin-converting enzyme (ACE) is a ubiquitous membrane ectoprotein found in mammalian tissues, and germinal ACE (gACE, also called testicular ACE) is found exclusively in male germinal cells after meiosis. Germinal ACE knockout in mice caused a defect in sperm binding to the zona pellucida of the oocyte^58^. Li *et al*. reported that the absence of gACE expression is responsible for fertilization failure^59^. Sperm mitochondrial-associated cysteine-rich protein (SMCP) is a constituent of the keratinous capsule surrounding sperm mitochondria that enhances sperm motility. Deletion of SMCP impaired sperm motility, resulting in sperm that fail to migrate in the female reproductive tract and penetrate the egg membranes during fertilization^60^. In view of the decreased expressions of SPACA1, ACE and SMCP in rat testis, it is proposed that arsenic affects fertilization by inhibiting the binding and fusion of sperm to egg.

### Arsenic induces male reproductive toxicity through the ERK/AKT/NF-κB-dependent pathway

Mitogen-activated protein kinases (MAPKs) are key regulatory proteins in cell signaling and participate in diverse functions, which are activated in response to a myriad of external stimuli. Previous studies have shown that numerous male reproductive functions, including spermatogenesis, sperm and Sertoli cell function are modulated via MAPK signaling pathways (e.g. extracellular signal-regulated kinases, ERKs)^61^. It was suggested that activated ERK1/2 (MAPK3/1) signaling impairs Sertoli cell function and increases germ cell apoptosis in mouse testes^62^,^63^. Protein kinase B (AKT) is an important regulator of cell growth, survival, proliferation, inflammatory and immune reaction in response to oxidative stress. PM2.5 exposure induced oxidative stress via phosphatidylinositol 3-kinase (PI3K)/AKT signaling pathway, which then impaired male reproduction in rats^64^. MAPK and AKT pathways act directly to phosphorylate nuclear factor kappa B (NF-κB) subunits to affect the ability of NF-κB to bind DNA and increase the transactivation of NF-κB-dependent genes^65^. NF-κB is involved in spermatogenesis via regulation of cellular apoptosis and Sertoli cell function in the testis, and the activation of NF-κB leads to defective spermatogenesis in mice and humans^66^,^67^. In addition, arsenic exposure has been reported to trigger ERK, PI3K/AKT and NF-κB signaling pathways in different cells^68^,^69^. In our study, the differential proteins and metabolites related to male reproduction were also observed to be involved in ERK/AKT/NF-κB pathway (Fig. 5). Together with the up-regulation of ERK1/2, PI3K, AKT, IKKγ and NFKB gene expression, as well as enhanced phosphorylated ERK/AKT level in rat testis (Fig. 6), we propose that arsenic may elicit male reproductive toxicity through activated ERK/AKT/NF-κB pathway.

**Figure 7:**
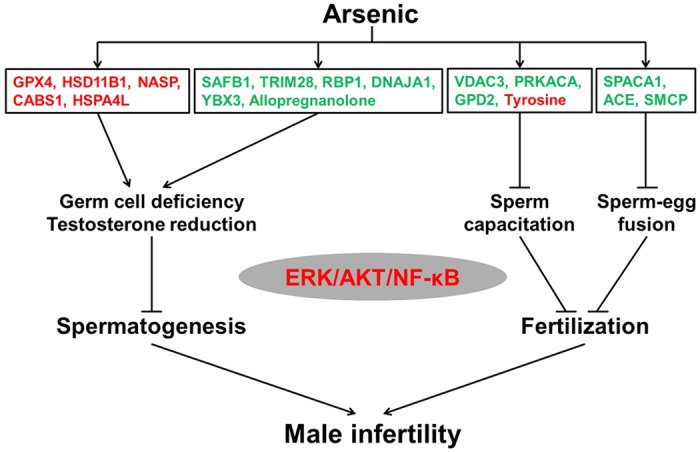
Schematic overview of the pathways through which arsenic induces male reproductive toxicity in rat testis. Molecules in red represent up-regulation, while the ones in green represent down-regulation.

In conclusion, through a combined proteomic and metabolomic analysis, the present study investigated the effects of arsenic exposure on the proteomic expression and metabolic pathways in rat testis. A series of differential proteins and metabolites specially related to male reproduction were identified. In addition, we suggest that the dysregulation of 16 proteins and 2 metabolites induced by arsenic would impair spermatogenesis and fertilization processes, which was involved in the activation of ERK/AKT/NF-κB-dependent pathway (Fig. 7). However, further study is still needed to reveal the mechanisms by which the arsenic-modulated proteins and metabolites interact with ERK/AKT/NF-κB signaling.

## Additional Information

**How to cite this article**: Huang, Q. *et al*. Integrated proteomics and metabolomics analysis of rat testis: Mechanism of arsenic-induced male reproductive toxicity. *Sci. Rep.* **6**, 32518; doi: 10.1038/srep32518 (2016).

## Supplementary Material

Supplementary Information

## Figures and Tables

**Table 1:** Differentially expressed proteins associated with reproductive process in rat testis exposed to arsenic.

Protein ID	Protein name	Gene name	Change	Fold change (Treatment/Control)^a^		
				1 mg/L	5 mg/L	25 mg/L
Q9R1Z0	Voltage-dependent anion-selective channel protein 3	Vdac3	↓	0.92 ± 0.02	0.85 ± 0.06	0.74 ± 0.15^**^
P27791	cAMP-dependent protein kinase catalytic subunit alpha	Prkaca	↓	0.73 ± 0.08^**^	0.79 ± 0.13^*^	0.79 ± 0.09^*^
B4F772	Heat shock 70 kDa protein 4L	Hspa4l	↑	1.33 ± 0.04^**^	1.21 ± 0.07^**^	1.27 ± 0.06^**^
D3Z9F9	Sperm acrosome membrane-associated protein 1	Spaca1	↓	0.79 ± 0.02^**^	0.81 ± 0.02^**^	0.76 ± 0.08^*^
P15205	Microtubule-associated protein 1B	Map1b	↓	0.66 ± 0.07^*^	0.64 ± 0.11^**^	0.70 ± 0.47^*^
P36970	Glutathione peroxidase 4	Gpx4	↑	1.03 ± 0.02	1.19 ± 0.08^*^	1.29 ± 0.07^**^
O88453	Scaffold attachment factor B1	Safb1	↓	0.81 ± 0.09	0.72 ± 0.22^*^	0.75 ± 0.07^*^
O08629	Transcription intermediary factor 1-beta	Trim28	↓	1.04 ± 0.05	0.86 ± 0.08^**^	0.79 ± 0.01^**^
P02696	Retinol-binding protein 1	Rbp1	↓	0.71 ± 0.12^**^	0.67 ± 0.08^**^	0.73 ± 0.06^*^
P16232	Corticosteroid 11-beta-dehydrogenase isozyme 1	Hsd11b1	↑	1.25 ± 0.04^*^	1.42 ± 0.13^**^	1.23 ± 0.04^*^
P21708	Mitogen-activated protein kinase 3	Mapk3	↑	0.94 ± 0.01	1.28 ± 0.06^**^	1.37 ± 0.03^**^
P35571	Glycerol-3-phosphate dehydrogenase, mitochondrial	Gpd2	↓	0.75 ± 0.09	0.68 ± 0.29^*^	0.6 ± 0.21^*^
P47820	Angiotensin-converting enzyme	Ace	↓	1.08 ± 0.03	0.88 ± 0.02^**^	0.72 ± 0.05^**^
P55063	Heat shock 70 kDa protein 1-like	Hspa1l	↑	1.12 ± 0.02^**^	1.09 ± 0.00^**^	1.20 ± 0.04^**^
P63036	DnaJ homolog subfamily A member 1	Dnaja1	↓	0.7 ± 0.04^**^	0.63 ± 0.17^**^	0.75 ± 0.13^*^
Q62764	Y-box-binding protein 3	Ybx3	↓	0.7 ± 0.16^*^	0.72 ± 0.13^*^	0.59 ± 0.23^**^
Q64298	Sperm mitochondrial-associated cysteine-rich protein	Smcp	↓	0.82 ± 0.01	0.7 ± 0.33	0.65 ± 0.24^*^
Q66HD3	Nuclear autoantigenic sperm protein	Nasp	↑	1.19 ± 0.02^*^	1.14 ± 0.10^*^	1.22 ± 0.05^**^
Q68FX6	Calcium-binding and spermatid-specific protein 1	Cabs1	↑	1.18 ± 0.03^*^	1.48 ± 0.11^**^	1.49 ± 0.06^**^

^a^The change of protein expression level was expressed as a treated/control ratio (control = 1, mean ± SD). A value >1 represents up-regulation whereas a value <1 indicates down-regulation. ^*^p < 0.05, ^**^p < 0.01.

**Table 2:** Differential metabolites in rat testis responsive to arsenic exposure.

HMDB ID	Metabolite	Measured MW (Da)	Change	Fold change (Treatment/Control)^a^		
				1 mg/L	5 mg/L	25 mg/L
HMDB00687	L-Leucine	132.1019	↑	0.94 ± 0.08	0.99 ± 0.10	1.82 ± 0.46^**^
HMDB00157	Hypoxanthine	137.0456	↓	0.80 ± 0.15^*^	0.74 ± 0.10^**^	0.48 ± 0.10^**^
HMDB00875	Trigonelline	138.0502	↑	4.28 ± 3.15	2.67 ± 2.56	3.65 ± 2.81^*^
HMDB00696	L-Methionine	150.058	↑	0.91 ± 0.14	0.98 ± 0.13	1.40 ± 0.20^**^
HMDB00159	L-Phenylalanine	166.0859	↑	0.98 ± 0.18	1.04 ± 0.16	2.20 ± 0.83^**^
HMDB12247	L-2,3-Dihydrodipicolinate	170.0323	↓	0.96 ± 0.09	0.95 ± 0.06	0.88 ± 0.11^*^
HMDB00158	L-Tyrosine	182.0808	↑	0.91 ± 0.15	0.96 ± 0.15	1.47 ± 0.26^**^
HMDB12150	2-Keto-6-acetamidocaproate	188.0703	↑	1.01 ± 0.20	1.12 ± 0.14	1.29 ± 0.40^**^
HMDB00201	L-Acetylcarnitine	204.1052	↑	1.23 ± 0.17^**^	1.22 ± 0.18^**^	3.21 ± 1.78^**^
HMDB00195	Inosine	269.0874	↓	0.80 ± 0.14^*^	0.74 ± 0.12^**^	0.47 ± 0.11^**^
HMDB01449	Allopregnanolone	319.2627	↓	0.78 ± 0.20	0.71 ± 0.41	0.36 ± 0.28^**^
HMDB03128	Cortolone	367.2601	↓	0.64 ± 0.27	0.56 ± 0.25^*^	0.30 ± 0.21^**^
HMDB11487	LysoPE(0:0/20:4(5Z,8Z,11Z,14Z))	502.2921	↑	1.80 ± 0.86	1.47 ± 0.38^*^	1.96 ± 0.90^*^

^a^The change of metabolite abundance is expressed as the average ratio of treatment/control (control = 1, mean ± SD). A value >1 represents up-regulation, whereas a value <1 indicates down-regulation. ^*^p < 0.05, ^**^p < 0.01.
