# Supplementary material for: Integrated proteomics and metabolomics analysis of rat testis: Mechanism of arsenic-induced male reproductive toxicity
Source: Sci Rep. 2016 Sep 2;6:32518. doi: 10.1038/srep32518 (PMC5009432; doi:10.1038/srep32518)
Supplement: Supplementary Information [file srep32518-s1.docx]

**Additional Information**

**Integrated proteomics and metabolomics analysis of rat testis: Mechanism of arsenic-induced male reproductive toxicity**

Qingyu Huang, Lianzhong Luo, Ambreen Alamdar, Jie Zhang, Liangpo Liu, Meiping Tian, Syed Ali Musstjab Akber Shah Eqani, Heqing Shen

Number of pages: 16

Number of tables: 7

Number of figures: 3

**Materials and methods**

**Label-free quantitative proteomics analysis**

Protein extraction and digestion

After thawed, 100 mg of testis tissue were homogenized in extraction buffer (50 mM Tris, 0.1% SDS, 10 mM HEPES, pH 8.1). After 30 min of extraction on ice, centrifugation was performed at 12 000 ×*g* for 15 min to remove the unlysed debris. The protein extracts were then precipitated with trichloroacetic acid/acetone and maintained at -20 °C overnight. After centrifugation at 12 000 ×*g* for 30 min, the pellets were resuspended in lysis buffer (6 M Urea, 2 M Thiourea, 20 mM DTT, 30 mM Tris, 0.1% SDS) and centrifuged at 12 000 ×*g* for 30 min. The supernatants were then collected, and protein content was measured using the Bradford assay.

Fifty microgram of protein sample was reduced by 10 mM dithiothreitol and alkylated by 40 mM iodoacetamide. Before tryptic digestion, 50 mM ammonium bicarbonate buffer was added to reduce the concentration of urea to 1 M. For in-solution digestion, trypsin was added to the protein mixture at an enzyme-to-substrate ratio of 1:50 (w/w). After incubation at 37 °C for 16 h, additional trypsin (1:100, w/w) was added to the sample and incubation was continued for 3 h to ensure complete digestion. C18 ZipTips were used to clean the tryptic peptides before nano-UPLC/MS/MS analysis.

Nano-UPLC/MS/MS analysis

Nano-UPLC/MS/MS analysis was performed on a Waters nanoACQUITY UPLC system (Waters, Milford, MA, USA) coupled to a Q Exactive mass spectrometer (Thermo Fisher, USA). The trapping was performed on a nanoACQUITY UPLC 2G-VIMTrap C18 column (5 μm, 20 mm × 180 μm i.d.), whereas elution was performed on an nanoACQUITY UPLC BEH130 C18 column (1.7 μm, 100 mm × 100 μm i.d.) (Waters, Milford, MA, USA). The mobile phases were (A) 98% acetonitrile with 0.1% formic acid and (B) 2% acetonitrile with 0.1% formic acid; the elution was carried out by following procedure: 0 min, 3% A; 25 min, 8% A; 135 min, 35% A; 145 min, 65% A; 180 min, 100% A. The flow rate was 0.3 µL/min and the injection volume of peptide sample was 5 µL.

Online electrospray of the eluting peptides into the Q Exactive mass spectrometer was achieved with the Easy Spray ion source (Thermo Fisher Scientific, USA). Full MS spectra were recorded at a resolution of 70,000 over a mass range of 350 to 1800 *m/z*. The automatic gain control target was set to 1×10^6^ and a maximum injection time of 50 ms was allowed. The 15 most intense peptide ions were chosen for fragmentation. The MS/MS spectra were recorded at a resolution of 17500 with the automatic gain control target set to 1×10^5^ and a maximum injection time of 100 ms. A mass window of 2.0 *m/z* was applied to precursor selection. Normalized collisional energy for the higher-energy collision-induced dissociation fragmentation was set to 28%. A dynamic exclusion with a time window of 45 s was applied. Singly charged molecules were not selected for fragmentation and the monoisotopic precursor selection was enabled. The underfill ratio (minimum percentage of the estimated target value at maximum fill time) was set to 1%. Triplicate analyses were performed for each protein sample.

**Metabolomics analysis**

Sample preparation

One hundred and fifty milligram of testis tissue was homogenized in 50% methanol, and an equal volume of 100% methanol was further added to precipitate the proteins. After sonication (5 min each time, 3 times), the homogenates were centrifuged at 16000 ×*g* for 15 min. The supernatants were dried using a Speedvac concentrator (Thermo Fisher Scientific, NC, USA), and then reconstituted with 100 μL of 10% methanol. The samples were centrifuged at 12000 ×*g* for 15 min, and the supernatants were collected for UPLC/MS analysis. A quality control (QC) sample was prepared by mixing aliquots of each sample, being broadly representative of the whole sample set.

UPLC/MS analysis

Metabolic profiling was conducted using a Waters ACQUITY UPLC system (Waters, Milford, MA, USA) coupled to a Q Exactive mass spectrometer (Thermo Fisher, USA). Chromatographic separation was performed on an ACQUITY UPLC BEH C18 column (1.7 μm, 100 mm × 2.1 mm i.d.) (Waters, Milford, MA, USA). For each sample, the run time was 20 min at a flow rate of 0.35 mL/min. The mobile phases were (A) methanol with 0.1% formic acid and (B) H_2_O with 0.1% formic acid. The programmed gradient was 0 min, 0% A; 6 min, 25% A; 10 min, 80% A; 12 min, 100% A; 15 min, 100% A; 15.5 min, 0% A; and 20 min, 0% A. The column was maintained at 50 °C and the injection volume was 5 μL.

A mass spectrometer equipped with heated electrospray ionization (HESI) source was operated in positive or negative ion mode with a scan range of 100 to 1000 *m/z*. Spray voltage was set at 3500 V for positive mode and 2500 V for negative mode. Probe heater temperature was set at 420 °C, and capillary temperature was set at 260 °C. Nitrogen gas was used as carrier gas. The flow rates of sheath gas, aux gas and sweep gas were 50, 10 and 1 L/min, respectively. Data were collected in centroid mode. All the samples were run in a randomized fashion to remove possible uncertainties from artifact-related injection order and gradual changes of instrument sensitivity in batch runs. One QC sample was injected at the start of the analytical batch, followed by analysis at every tenth sample injection throughout the running sequence. MS/MS mode was used to identify potential biomarkers with argon as collision gas. MS/MS was performed by normalized collision energy (NCE) technology with 30% NCE for the biomarkers with 17500 resolution and isolation window *m/z* of 1.0.

Data processing

UPLC-MS data were processed with SIEVE software (Thermo Fisher Scientific, NC, USA) to generate a two-dimensional data table of ion peaks (*m/z*-retention time pairs) and their respective intensities (peak areas). Peak detection, retention time correction and alignment were performed using the following parameters: mass range of 100–1000 *m/z*, mass tolerance of 20 ppm, retention time (RT) range of 0.5–17 min, and RT width threshold of 0.5 min. All data in the table were normalized to total intensities to eliminate systematic bias, and any variables with missing values in more than 20% of the samples were excluded.

**Table S1.** MS/MS information of identified differential metabolites

| HMDB ID | Metabolite | Measured  MW (Da) | MS/MS fragments |
| --- | --- | --- | --- |
| HMDB00687 | L-Leucine | 132.1019 | 86(21), 132(100) |
| HMDB00157 | Hypoxanthine | 137.0456 | 94(11), 119(100), 137(3) |
| HMDB00875 | Trigonelline | 138.0502 | 52(13), 77(7), 95(76), 138(100) |
| HMDB00696 | L-Methionine | 150.058 | 104(40), 133(100) |
| HMDB00159 | L-Phenylalanine | 166.0859 | 103(94), 120(14) |
| HMDB12247 | L-2,3-Dihydrodipicolinate | 170.0323 | 152(60), 170(100) |
| HMDB00158 | L-Tyrosine | 182.0808 | 119(28), 123(35), 147(100) |
| HMDB00201 | L-Acetylcarnitine | 204.1052 | 57(21), 60(10) |
| HMDB00195 | Inosine | 269.0874 | 137(100) |

**Table S2.** Sequences of the specific primers for real-time PCR

| Gene name | Primer sequence (5’→3’) |
| --- | --- |
| ERK1 | F: GCACGCTGAGAGAAATCCAG  R: TGTATGTACTTGAGGCCCCG |
| ERK2 | F: CTATTTGCTTTCTCTCCCGCA  R: GCCAGAGCCTGTTCAACTTC |
| PI3K | F: TTGGTCTGTGTCCCGAGAAG  R: TGTGGCTCCATAGGAAGTCT |
| AKT | F: CCACGCTACTTCCTCCTCAA  R: GATGATGAAGGTGTTGGGCC |
| IKKγ | F: CAGCCACATTAAGAGCAGCA  R: AAAGCTCCTTCCTCTCCACC |
| NFKB | F: CGCCACCGGATTGAAGAAAA  R: TTGATGGTGCTGAGGGATGT |
| β-ACTIN | F: TGTGTTGTCCCTGTATGCCT  R: AATGTCACGCACGATTTCCC |

**Table S3.** Body weight and testicular coefficient of rats after exposure to arsenic

| Group | Body weight (BW, g) | Testis weight (TW, g) | Testicular coefficient (TW/BW, %) |
| --- | --- | --- | --- |
| Control | 586.60±29.36 | 1.81±0.16 | 0.31±0.03 |
| 1 mg/L | 633.00±21.18 | 1.93±0.16 | 0.30±0.03 |
| 5 mg/L | 621.67±47.32 | 1.92±0.12 | 0.31±0.03 |
| 25 mg/L | 590.36±36.14 | 1.74±0.19 | 0.30±0.03 |

**Table S5.** Differentially expressed proteins in rat testis following arsenic exposure

| No. | Protein ID | Protein name | Gene name | Change | Fold change (Treatment/Control)*^a^* | | |
| --- | --- | --- | --- | --- | --- | --- | --- |
|  |  |  |  |  | 1 mg/L | 5 mg/L | 25 mg/L |
| 1 | Q9R1Z0 | Voltage-dependent anion-selective channel protein 3 | Vdac3 | ↓ | 0.92±0.02 | 0.85±0.06 | 0.74±0.15** |
| 2 | P27791 | cAMP-dependent protein kinase catalytic subunit alpha | Prkaca | ↓ | 0.73±0.08* | 0.79±0.13* | 0.79±0.09* |
| 3 | Q6I8Q6 | Histone H2A | Hist1h2af | ↓ | 0.91±0.04 | 0.67±0.09** | 0.63±0.04** |
| 4 | B0BN47 | Glutathione S-transferase, mu 6 | Gstm6 | ↓ | 1.05±0.15 | 0.91±0.10 | 0.69±0.09* |
| 5 | B0BN85 | Suppressor of G2 allele of SKP1 homolog | Sugt1 | ↑ | 1.12±0.08 | 1.15±0.04 | 1.24±0.08* |
| 6 | B4F772 | Heat shock 70 kDa protein 4L | Hspa4l | ↑ | 1.33±0.04** | 1.21±0.07** | 1.27±0.06** |
| 7 | D3Z9F9 | Sperm acrosome membrane-associated protein 1 | Spaca1 | ↓ | 0.79±0.02** | 0.81±0.02** | 0.76±0.08* |
| 8 | D3ZB81 | ADP/ATP translocase 4 | Slc25a31 | ↑ | 1.30±0.16* | 1.51±0.05** | 1.75±0.12** |
| 9 | D3ZMS1 | Splicing factor 3B subunit 2 | Sf3b2 | ↑ | 1.12±0.07 | 1.25±0.07 | 1.40±0.06** |
| 10 | Q00715 | Histone H2B | Hist1h2bl | ↓ | 0.84±0.05* | 0.83±0.06* | 0.79±0.10* |
| 11 | D4A720 | Serine/arginine-rich splicing factor 7 | Srsf7 | ↓ | 0.99±0.05 | 0.90±0.11 | 0.79±0.08* |
| 12 | Q641Y0 | Dolichyl-diphosphooligosaccharide--protein glycosyltransferase 48 kDa subunit | Ddost | ↓ | 0.76±0.09* | 0.83±0.10 | 0.75±0.07* |
| 13 | P15205 | Microtubule-associated protein 1B | Map1b | ↓ | 0.66±0.07* | 0.64±0.11** | 0.70±0.47* |
| 14 | Q62826 | Heterogeneous nuclear ribonucleoprotein M | Hnrnpm | ↑ | 1.26±0.11** | 1.52±0.05** | 1.34±0.05** |
| 15 | F1M8X9 | Prostaglandin E synthase 2 | Gbf1 | ↑ | 1.09±0.03 | 1.21±0.19 | 1.27±0.12* |
| 16 | Q6P7A2 | Ubiquitin conjugation factor E4 A | Ube4a | ↑ | 1.06±0.08 | 1.26±0.17 | 1.30±0.02* |
| 17 | P36970 | Glutathione peroxidase 4 | Gpx4 | ↑ | 1.03±0.02 | 1.19±0.08* | 1.29±0.07** |
| 18 | P17132 | Heterogeneous nuclear ribonucleoprotein D | Hnrpd | ↑ | 0.88±0.09 | 0.96±0.03 | 1.26±0.03** |
| 19 | Q62812 | Myosin-9 | Myh9 | ↓ | 0.35±0.04** | 0.23±0.06** | 0.45±0.04** |
| 20 | Q6AYX5 | Outer dense fiber protein 2 | Odf2 | ↑ | 1.37±0.02** | 1.21±0.07** | 1.32±0.03** |
| 21 | G3V852 | Talin-1 | Tln1 | ↓ | 1.01±0.02 | 0.87±0.07 | 0.75±0.03* |
| 22 | P31000 | Vimentin | Vim | ↓ | 0.98±0.04 | 0.89±0.04** | 0.80±0.01** |
| 23 | G3V9R8 | Heterogeneous nuclear ribonucleoprotein C | Hnrnpc | ↑ | 1.14±0.01* | 1.34±0.10** | 1.25±0.00** |
| 24 | Q4KLZ3 | DAZ associated protein 1 | Dazap1 | ↑ | 1.39±0.06** | 1.36±0.04** | 1.25±0.12* |
| 25 | O88453 | Scaffold attachment factor B1 | Safb1 | ↓ | 0.81±0.09 | 0.72±0.22* | 0.75±0.07* |
| 26 | Q6PEC1 | Tubulin-specific chaperone A | Tbca | ↑ | 1.44±0.11** | 1.29±0.06* | 1.29±0.08* |
| 27 | O08629 | Transcription intermediary factor 1-beta | Trim28 | ↓ | 1.04±0.05 | 0.86±0.08** | 0.79±0.01** |
| 28 | P02696 | Retinol-binding protein 1 | Rbp1 | ↓ | 0.71±0.12** | 0.67±0.08** | 0.73±0.06* |
| 29 | P02793 | Ferritin light chain 1 | Ftl1 | ↓ | 0.93±0.11 | 0.72±0.11* | 0.68±0.06** |
| 30 | P04906 | Glutathione S-transferase P | Gstp1 | ↓ | 1.03±0.08 | 0.82±0.04* | 0.69±0.02** |
| 31 | P10888 | Cytochrome c oxidase subunit 4 isoform 1, mitochondrial | Cox4i1 | ↑ | 1.01±0.15 | 1.00±0.14 | 1.28±0.08* |
| 32 | P12785 | Fatty acid synthase | Fasn | ↓ | 0.70±0.08** | 0.72±0.09** | 0.79±0.05* |
| 33 | P13086 | Succinyl-CoA ligase [ADP/GDP-forming] subunit alpha, mitochondrial | Suclg1 | ↑ | 1.15±0.10 | 1.09±0.07 | 1.28±0.13* |
| 34 | P14668 | Annexin A5 | Anxa5 | ↓ | 0.94±0.03 | 0.86±0.05** | 0.76±0.00** |
| 35 | P16232 | Corticosteroid 11-beta-dehydrogenase isozyme 1 | Hsd11b1 | ↑ | 1.25±0.04* | 1.42±0.13** | 1.23±0.04* |
| 36 | P21708 | Mitogen-activated protein kinase 3 | Mapk3 | ↑ | 0.94±0.01* | 1.28±0.06** | 1.37±0.03** |
| 37 | P32551 | Cytochrome b-c1 complex subunit 2, mitochondrial | Uqcrc2 | ↑ | 1.41±0.10** | 1.23±0.10* | 1.22±0.07* |
| 38 | P35571 | Glycerol-3-phosphate dehydrogenase, mitochondrial | Gpd2 | ↓ | 0.75±0.09 | 0.68±0.29* | 0.6±0.21* |
| 39 | P47820 | Angiotensin-converting enzyme | Ace | ↓ | 1.08±0.03* | 0.88±0.02** | 0.72±0.05** |
| 40 | P47942 | Dihydropyrimidinase-related protein 2 | Dpysl2 | ↓ | 0.84±0.06 | 0.88±0.07 | 0.80±0.07* |
| 41 | P48037 | Annexin A6 | Anxa6 | ↓ | 0.95±0.06 | 0.85±0.06* | 0.79±0.03** |
| 42 | P51647 | Retinal dehydrogenase 1 | Aldh1a1 | ↓ | 0.88±0.08 | 0.83±0.07* | 0.70±0.03** |
| 43 | P55063 | Heat shock 70 kDa protein 1-like | Hspa1l | ↑ | 1.12±0.02** | 1.09±0.00** | 1.20±0.04** |
| 44 | P63036 | DnaJ homolog subfamily A member 1 | Dnaja1 | ↓ | 0.7±0.04** | 0.63±0.17** | 0.75±0.13* |
| 45 | P85968 | 6-phosphogluconate dehydrogenase, decarboxylating | Pgd | ↓ | 0.79±0.01** | 0.78±0.04** | 0.82±0.03** |
| 46 | Q00729 | Histone H2B type 1-A | Hist1h2ba | ↓ | 0.80±0.08 | 0.82±0.06 | 0.79±0.09* |
| 47 | Q02874 | Core histone macro-H2A.1 | H2afy | ↓ | 0.83±0.08* | 0.68±0.07** | 0.76±0.01** |
| 48 | Q07936 | Annexin A2 | Anxa2 | ↓ | 0.70±0.01** | 0.65±0.04** | 0.56±0.05** |
| 49 | Q498E3 | Protein LOC290876 | LOC290876 | ↑ | 1.25±0.20 | 0.99±0.13 | 1.39±0.11* |
| 50 | Q4QR73 | DnaJ (Hsp40) homolog, subfamily A, member 4 | Dnaja4 | ↑ | 1.33±0.06** | 1.39±0.02** | 1.36±0.05** |
| 51 | Q5I0H9 | Protein disulfide-isomerase A5 | Pdia5 | ↑ | 1.18±0.13* | 1.03±0.06 | 1.28±0.06** |
| 52 | Q5XIJ9 | Succinyl-CoA:3-ketoacid coenzyme A transferase 2A, mitochondrial | Oxct2a | ↑ | 1.06±0.04 | 1.25±0.03** | 1.27±0.01** |
| 53 | Q5XXR3 | Rho guanine nucleotide exchange factor 6 | Arhgef6 | ↑ | 1.22±0.06** | 1.06±0.04 | 1.25±0.10** |
| 54 | Q62669 | Hemoglobin subunit beta-1 | Hbb-b1 | ↑ | 0.99±0.06 | 1.28±0.08** | 1.24±0.06** |
| 55 | Q62764 | Y-box-binding protein 3 | Ybx3 | ↓ | 0.7±0.16* | 0.72±0.13* | 0.59±0.23** |
| 56 | Q64298 | Sperm mitochondrial-associated cysteine-rich protein | Smcp | ↓ | 0.82±0.01 | 0.7±0.33 | 0.65±0.24* |
| 57 | Q66HD3 | Nuclear autoantigenic sperm protein | Nasp | ↑ | 1.19±0.02* | 1.14±0.10* | 1.22±0.05** |
| 58 | Q66HF1 | NADH-ubiquinone oxidoreductase 75 kDa subunit, mitochondrial | Ndufs1 | ↑ | 0.93±0.03 | 1.10±0.02 | 1.23±0.08** |
| 59 | Q68FX6 | Calcium-binding and spermatid-specific protein 1 | Cabs1 | ↑ | 1.18±0.03* | 1.48±0.11** | 1.49±0.06** |
| 60 | Q68FY0 | Cytochrome b-c1 complex subunit 1, mitochondrial | Uqcrc1 | ↑ | 1.22±0.04** | 1.09±0.10 | 1.22±0.01** |
| 61 | Q6AXX6 | Redox-regulatory protein FAM213A | Fam213a | ↑ | 0.91±0.09 | 1.23±0.11* | 1.23±0.01* |
| 62 | Q6AYF9 | Interferon-inducible GTPase 5 | Irgc | ↑ | 1.15±0.02 | 1.24±0.04* | 1.34±0.13** |
| 63 | Q6AYJ1 | ATP-dependent DNA helicase Q1 | Recql | ↑ | 1.19±0.02 | 1.51±0.13** | 1.32±0.10* |
| 64 | Q6IMY8 | Heterogeneous nuclear ribonucleoprotein U | Hnrnpu | ↑ | 1.06±0.10 | 1.26±0.03** | 1.28±0.04** |
| 65 | Q6P9U0 | Serpin B6 | Serpinb6 | ↓ | 0.71±0.10* | 0.79±0.14 | 0.68±0.01** |
| 66 | P08430 | UDP-glucuronosyltransferase 1-6 | Ugt1a6 | ↑ | 1.29±0.08** | 1.30±0.03** | 1.25±0.02** |
| 67 | Q9QXQ0 | Alpha-actinin-4 | Actn4 | ↓ | 0.99±0.08 | 0.79±0.02* | 0.70±0.02** |
| 68 | Q9WVJ6 | Protein-glutamine gamma-glutamyltransferase 2 | Tgm2 | ↓ | 0.95±0.08 | 0.80±0.06* | 0.75±0.10* |
| 69 | Q9Z1B2 | Glutathione S-transferase Mu 5 | Gstm5 | ↑ | 1.18±0.05** | 1.14±0.04* | 1.23±0.07** |
| 70 | Q9Z272 | ARF GTPase-activating protein GIT1 | Git1 | ↑ | 1.07±0.08 | 1.14±0.06 | 1.26±0.06** |

*^a^* The change of protein expression level was expressed as a treated/control ratio (control =1, mean ± SD). A value >1 represents up-regulation whereas a value <1 indicates down-regulation. **p*<0.05, ***p*<0.01.

**Table S6.** Metabolic pathways significantly altered by arsenic in rat testis

| No. | Pathway | Total*^a^* | Hits*^b^* | *p* value*^c^* |
| --- | --- | --- | --- | --- |
| 1 | Phenylalanine, tyrosine and tryptophan biosynthesis | 4 | 2 | 3.99E-04 |
| 2 | Aminoacyl-tRNA biosynthesis | 67 | 4 | 1.77E-03 |
| 3 | Phenylalanine metabolism | 9 | 2 | 2.34E-03 |
| 4 | Ubiquinone and other terpenoid-quinone  biosynthesis | 3 | 1 | 2.55E-02 |

*^a^* The total number of compounds in the pathway.

*^b^* The number of compounds derived from metabolomics analysis.

*^c^* Probability of identified differential metabolite list associates to the generated pathway.

**Table S7.** Molecular networks involved in arsenic treatment in rat testis

| Network | Molecules in Network | Score | Focus Molecules | Top Diseases and Functions |
| --- | --- | --- | --- | --- |
| 1 | **ACE**, Akt, **allopregnanolone**, Alp, Ap1, CD3, Creb, **DNAJA1**, DNAJC6, epi-androsterone, ERK1/2, **GPD2**, **GPX4**, **HSD11B1**, HSP, Hsp70, **HSPA1L**, **HSPA4L**, IL1, Insulin, **L-tyrosine**, Lh, **MAPK3**, **NASP**, NFκB (complex), PI3K (complex), Pki, **PRKACA**, PROKR1, **RBP1**, TEX101, **TRIM28**, **VDAC3**, **YBX3**, ZNF354C | 45 | 16 | Reproductive System Development and Function, Organ Morphology,  Organismal Injury and Abnormalities |
| 2 | 75 NGF, AMBRA1, ANTXR1, ATG10, ATG48, Dynein, ERP44, GABRR1, IL12RB1, KIAA0368, MAG, **MAP1B**, MAP1LC3A, MELK, NBR1, PAFAH1B1, PPT1, PRKAG2, RASSF2, RASSF3, RASSF5, **SAFB**, SAFB2, SERPINE2, **SPACA1**, SRSF6, STRAP, TP53, TRA2B, TUBA8, TUBB3, TUBB2B, tubulin (family), VAPA, ZNF207 | 6 | 3 | Cancer,  Organismal Injury and Abnormalities, Reproductive System Disease |
| 3 | D-glucose, PAPOLB, **Smcp**, TAF7L | 3 | 1 | Reproductive System Development and Function,  Organ Morphology,  Cancer |
| 4 | C7orf43, **CABS1**, TRAPPC1, TRAPPC2, TRAPPC3, TRAPPC4, TRAPPC5, TRAPPC9, TRAPPC10, TRAPPC2L | 2 | 1 | Cancer, Gastrointestinal Disease, Organismal Injury and Abnormalities |


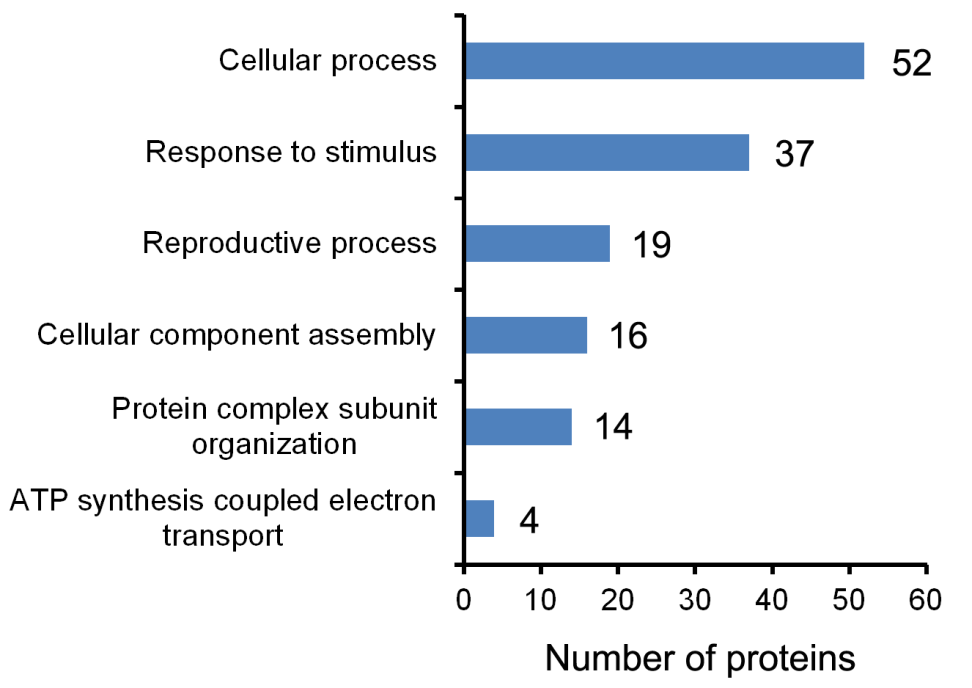


**Figure S1.** Functional distribution of the diﬀerentially expressed proteins in rat testis exposed to arsenic.


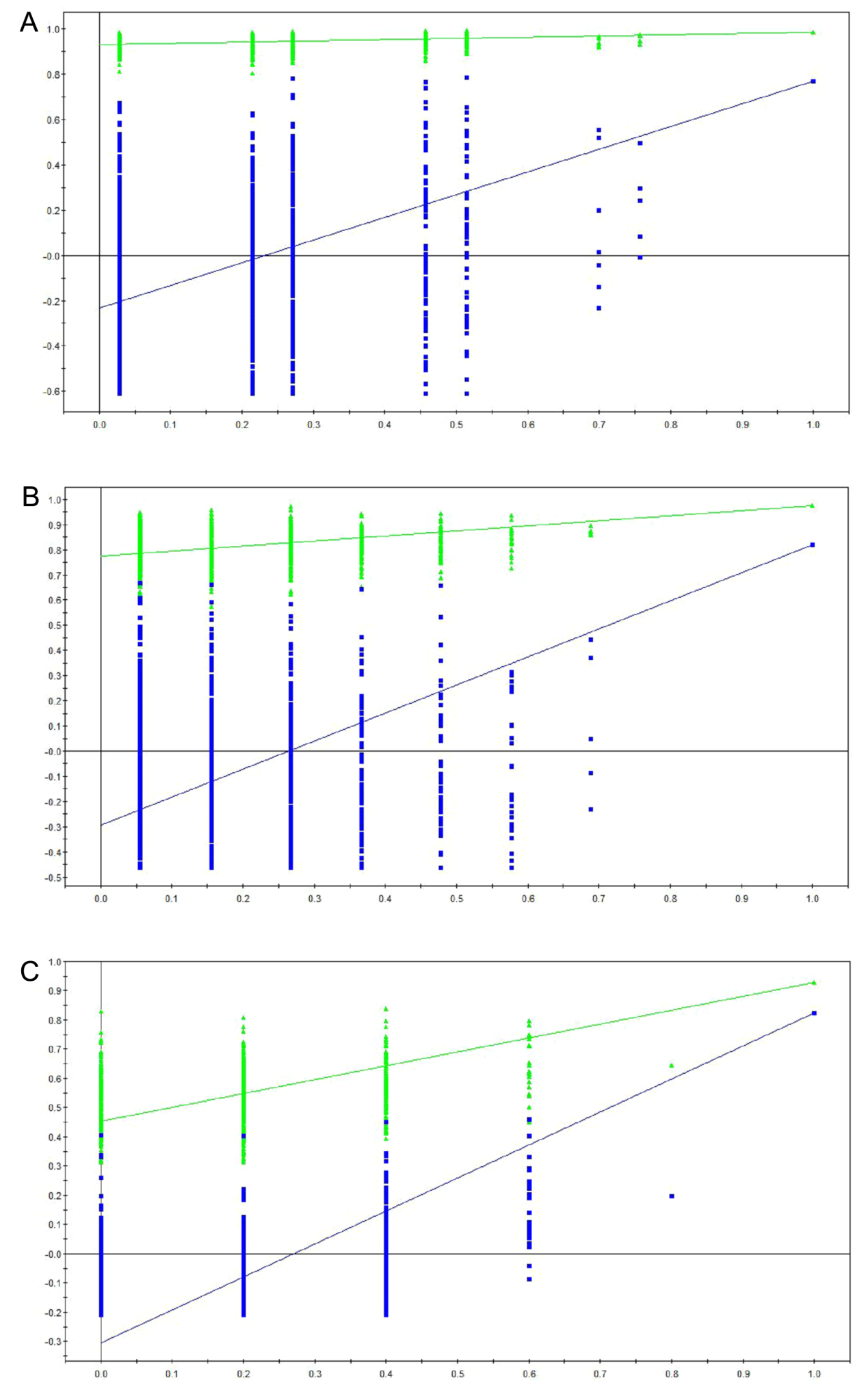


**Figure S2.** Random permutation test results (n = 999) of the PLS-DA model. R^2^ (green line) is the explained variance, and Q^2^ (blue line) is the predictive ability of the model. The calculated R^2^ and Q^2^ values were lower than the original ones in the validation plot, and the Q^2^ intercepted the vertical axis was below zero, therefore, the model was considered to be valid. (A) 1 mg/L; (B) 5 mg/L; (C) 25 mg/L


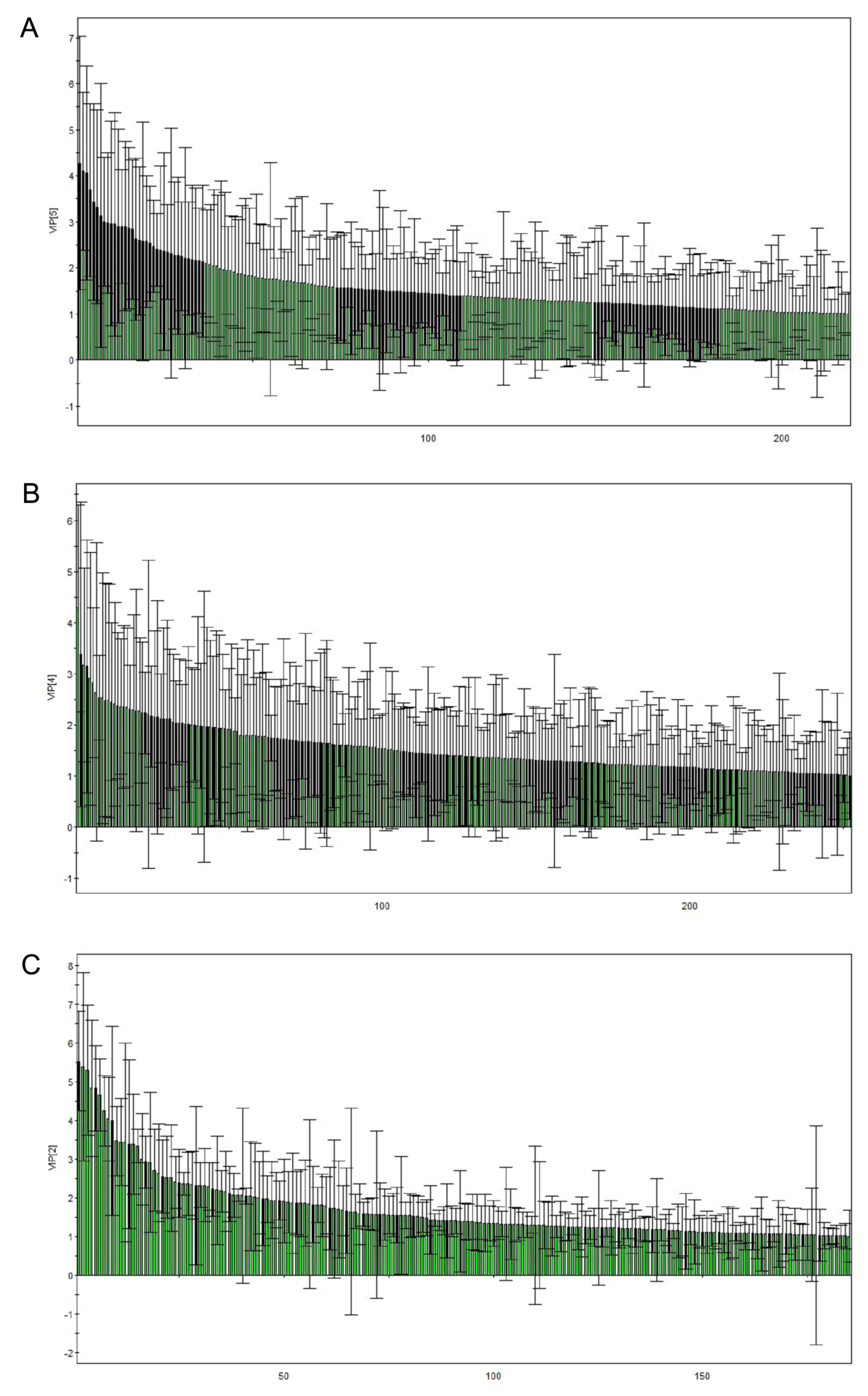


**Figure S3.** Variable inﬂuence in projection (VIP) plots of established PLS-DA model. The variables with negative jack-knifed conﬁdence intervals were removed from the potential biomarker list. (A) 1 mg/L; (B) 5 mg/L; (C) 25 mg/L
